# Seroprevalence of Hepatitis E Among Pregnant Women in Urmia, Iran

**DOI:** 10.5812/hepatmon.10931

**Published:** 2013-11-15

**Authors:** Zakieh Rostamzadeh Khameneh, Nariman Sepehrvand, Hamid-Reza Khalkhali

**Affiliations:** 1 Department of Microbiology and Immunology, Urmia University of Medical Sciences, Urmia, IR Iran; 2 Students’ Research Committee, Urmia University of Medical Sciences, Urmia, IR Iran; 3 Department of Epidemiology and Biostatistics, Urmia University of Medical Sciences, Urmia, IR Iran

**Keywords:** Pregnancy, Hepatitis E, Women, Enzyme-Linked Immunosorbent Assay

## Abstract

**Background:**

While hepatitis E virus (HEV) mostly causes self-limited disease in general population, it is more severe in pregnant women.

**Objectives:**

This study aimed to investigate the seroprevalence of anti-HEV IgG among a population of pregnant women in West Azerbaijan of Iran .

**Patients and Methods:**

One hundred thirty six pregnant women referred to urban health centers of Urmia for pursuing pregnancy-related health services were enrolled in a descriptive, cross-sectional study. Anti-HEV IgG antibody was evaluated using enzyme-linked immunosorbent assay (enzyme-linked immunosorbent assay, ELISA; Dia.Pro; Diagnostic Bioprobes).

**Results:**

Only five (3.6%) of 136 cases had positive results for anti-HEV IgG. There was no significant difference between age (P=0.88), and income level (P = 0.19) of the two seropositive and seronegative groups. All seropositive cases were from urban areas.

**Conclusions:**

The seroprevalence of anti-HEV IgG is low in the population of pregnant women in , similar to the rates reported from developed countries. Effective health services and provision of safe water supplies in Urmia may take role in this low prevalence rate.

## 1. Background

While hepatitis E virus (HEV) mostly causes a self-limited disease in developing countries, but the nature of disease in pregnant women is more severe (1). The etiologies of more severe presentation in pregnancy are under investigation yet. Associated hormonal changes (estrogen and progesterone) during pregnancy, reduced expression of progesterone receptor and progesterone induced blocking factor, a higher IL-12/IL-10 ratio, down-regulation of the P65 component of nuclear factor (NF-KappaB) with a predominant T-helper type2 (Th2) bias in the T-cell response along with host susceptibility factors, mediated by human leukocyte antigen expression, higher prevalence of folate deficiency in HEV in pregnant women of endemic areas, and a higher viral load in pregnancy due to the influence of sex hormones are some etiologies proposed for the worse prognosis of HEV infection in pregnancy (2-4). 

## 2. Objectives 

Since the prevalence of HEV infection has not been studied before in the population of Iranian pregnant women, we aimed to investigate the seroprevalence of anti-HEV IgG in a population of pregnant women in Urmia, Iran.

## 3. Patients and Methods

Approval was obtained from the Scientific and Ethical Review Board of Urmia University of Medical Sciences (UMSU), in Urmia, Iran. The minimum adequate sample size was calculated to be 126 using a predicted p of 9%, and a d of 0.05. One hundred and thirty six (136) pregnant women referred to urban health centers of Urmia for pursuing pregnancy-related health services were enrolled in this descriptive, cross-sectional study. 

According to the “Integrated Mothers’ Care Program”, All pregnant women in Iran should be covered by the health system via urban or rural health centers. They are visited and examined regularly by midwives and physicians during pregnancy, receiving necessary consults and complementary medications. In a convenient sampling method, we selected the first pregnant woman among every three women referred for pregnancy-related cares from five randomly selected urban health centers of Urmia, located in the center as well as four different directions of the city, during spring months. The sample size was distributed among centers according to the population size covered by each center. Informed consent was obtained from each subject prior to participation. 

Two milliliter blood samples were obtained via venipuncture for serological analyses. Samples were centrifuged, and sera were separated immediately. Sera were stored at –20 ºC, and tested for the presence of anti-HEV IgG antibody by enzyme-linked immunosorbent assay (enzyme-linked immunosorbent assay, ELISA; Dia.Pro; Diagnostic Bioprobes).

All collected data were analyzed using SPSS software ver16 (Chicago, IL). Descriptive statistics were reported as the mean ± SD for continuous variables and as the frequency (%) for dichotomous variables. To evaluate the association between different factors, we performed Chi-square analysis or Fisher’s exact test. Quantitative variables were compared using independent t-test. P < 0.05 was considered statistically significant.

## 4. Results

The mean age of 136 pregnant women was 25.12 ± 4.91 years (ranging from 14 to 39). 

Only five (3.6%; 95%CI: 1.34%-8.66%) of 136 cases had positive results for anti-HEV IgG using ELISA method. None of seropositive cases reported any history of blood transfusion, tattooing, or Hijama (a traditional medical practice for therapeutic purposes in ). Generally 20 cases had a history of abortion in their previous pregnancies (15 cases for one time, 2 for two times, 2 for three times, and one for 4 times). Only one HEV seropositive case reported an abortion in her previous pregnancy. Stillbirth was only reported in one HEV seronegative participant. 

There was no significant difference between age (P = 0.88), pregnancy times (P = 0.56), gestational ages (P = 0.88), and income level (P = 0.19) of the two seropositive and seronegative groups. The comparison of age, place of birth, place of residency, and educational level between the two seropositive and seronegative groups was demonstrated in Table 1. 

**Table 1.** Characteristics of Participants in the Two Seropositive and Seronegative Groups

Characteristics	HEV Seropositive, n = 5	HEV Seronegative, n = 131)	P value
**Age, mean (SD), y**	24.8 ± 4.6	25.1 ± 4.9	0.88
**Place of Birth**			0.06
Urban	5	68	
Rural	0	63	
**Place of Residence**			0.62
Urban	5	119	
Rural	0	12	
**Educational level**			0.62
Illiterate	3	46	
Primary	1	56	
Secondary	1	21	
Diploma and higher	0	8	
**Pregnancy times**			0.56
1	2	49	
2	1	44	
3	2	20	
> 4	0	17	
**Gestational Age, mean (SD), w**	24.8 ± 4.6	25.1 ± 4.9	0.88
**Income level, mean (SD), IR Rials/month**	8340000 ± 1520000	5380000 ± 2590000	0.19

## 5. Discussion

Iran, located in the Middle East is an endemic country for hepatitis E, with few suspected outbreaks of HEV (5). A population-based study in Iran reported a 9.6% prevalence rate of anti-HEV IgG among the healthy population (6). 

The seroprevalence of HEV was significantly low in pregnant women of Urmia. Indeed the rate of HEV seropositivity is not expected to be higher in pregnant women compared to general population, but the disease is demonstrated to be more severe with poorer prognosis in this specific population. 

Regarding the difference between our findings and the previous reports from Iran, the geographic distribution of disease could be different within the borders of a specific country. Various studies have reported different prevalence rates from Iran. Taremi et al. in 2007 reported a rate of 7.8% for anti-HEV seropositivity among healthy blood donors in Tabriz, located in East Azerbaijan province, a neighboring province to Urmia (7). Other study by Mohebbi et al. reported a higher rate (9.3%) in Tehran(8). Ataei and his associates in Isfahan reported this rate as 3.8% among the general population, and 4.2% among the female population (9). In the study of Saffar et al. in Sari city in northern Iran, the rate of anti-HEV positive cases was 2.3% among children and young adults (10). Our study in association with other reports from different regions of Iran may provide the required evidence for developing a prevalence map for HEV considering its geographical distribution.

The prevalence of anti-HEV IgG is considerably higher in Africa (from 15-30% in central Africa to 84.3% in Nile Delta of Egypt)(11, 12) and southern Asia (almost 30%)(13). But in the developed world this rate is significantly low. Lindemann et al. in a study on 1040 pregnant women in Spain reported the rate of anti-HEV IgG as 3.6%, which is similar to our findings (14). 

The prevalence rate of HEV infection in a population of pregnant women in Urmia is one of the lowest rates reported till now from Iran and the Middle East, and is closer to the prevalence rate in developed countries. The findings seem to be due to epidemiological reasons rather than methodology. It may be due to better sanitation, efficient health system and provision of safer water supplies in this city in comparison with other regions of Iran. Region’s geographical features and the season of sampling could be some other main reasons for the wide heterogeneity among different studies from Iran. Serum samples in our study were collected during spring months, but unfortunately most of the other studies did not mention the season of their sampling. Similar to the study of Oncu et al. (15), we did not find any significant correlation among age and HEV seropositivity. But Cevrioglu et al.reported a significant association between age and higher anti-HEV positive values (16). 

In our study, all the HEV seropositive cases were from urban areas. Begum et al. from India and Caron et al. from Gabon found a similar finding, and in their study exposure to HEV during pregnancy was higher in urban areas than rural populations (13, 17). 

In another study by Hannachi et al. in Tunisia, history of agricultural work, kind of water, sewage treatment, and contact with animals (which are all more related to the rural life rather than urban) were not correlated with the presence of anti-HEV antibody (11). But in contrast with these studies, Cevrioglu et al. from Turkey reported a significant correlation between rural residence and higher anti-HEV positive values (16). 

Since in our study all cases were selected randomly among pregnant women referred to public health centers of Urmia (missing the referrals to the private sector), our study population may lack a portion of pregnant women with higher socioeconomic and educational level. One the other hand, we used convenient sampling in this study. Although the data coming from a convenient sampling could be still valuable, but performing further studies with larger sample size and cluster random sampling would be more helpful.
